# Dual mode imaging in mid infrared with thermal signal reconstruction for innovative diagnostics of the “Monocromo” by Leonardo da Vinci

**DOI:** 10.1038/s41598-021-01837-8

**Published:** 2021-11-18

**Authors:** Claudia Daffara, Simone Parisotto, Paola Ilaria Mariotti, Dario Ambrosini

**Affiliations:** 1 Department of Computer Science, University of Verona, Strada le Grazie 15, 37134 Verona, Italy; 2 Department of Applied Mathematics and Theoretical Physics, University of Cambridge, Wilberforce Road, Cambridge, CB3 0WA UK; 3 Opificio delle Pietre Dure, V.le F. Strozzi, 1 (Fortezza da Basso), 50129 Firenze, Italy; 4 Department of Industrial and Information Engineering and Economics, University of L’Aquila, P.le Pontieri 1, 67100 Monteluco di Roio, AQ Italy

**Keywords:** Applied physics, Optical techniques, Environmental social sciences

## Abstract

Dual mode imaging in the mid infrared band, a joint use of thermography and quasi-thermal reflectography, was recently proposed as a full field diagnostic tool in cultural heritage. Here we discuss for the first time, to the best of our knowledge, a detailed application of such non destructive technique to the diagnostics of frescoes, with an emphasis on the location of detachments. We also investigate the use of a thermographic method based on TSR (thermal signal reconstruction), in a long pulse stimulus scheme, as well as the spatial registration of thermal images after post-processing analysis to their visible counterpart, so as to obtain a fine resolution diagnostic map. As an exemplar case study, we report about the application of dual mode imaging with a 500 ${\upmu }\hbox {m}$ pixel size at object plane on the “Monocromo”, a fresco by Leonardo da Vinci located in the Sforza Castle (Milan, Italy). Our technique was used to guide the conservators during the restoration works, opening new perspectives in artwork diagnostics.

## Introduction

Among the state-of-the-art nondestructive techniques applied to artworks^1–3^, infrared methods are of great importance. In particular, infrared thermography can be especially used to assist the restoration of mural paintings as it allows a remote and wide-field imaging of hidden features, such as structural defects and materials’ discontinuities^4–6^. Thermography in the LWIR (Long Wavelength InfraRed) region from 8 ${\upmu }\hbox {m}$ to 12 ${\upmu }\hbox {m}$ is very effective in the detection of in-depth alteration of the wall support^7^, but it is less suitable for some specific problems that require a sharp imaging; the most crucial one is the analysis of the detached areas of the pictorial paint and plaster layers^8^. The MWIR (Mid Wavelength InfraRed) region from 3 ${\upmu }\hbox {m}$ to 5 ${\upmu }\hbox {m}$, which is characterized by a lower diffraction limit and less affected by the ambient contribution, represents an alternative solution^9^. MWIR thermography has been applied to the analysis of different kinds of artifacts^10^. A recognized critical issue is that a fine resolution map requires to perform the mosaicking and the spatial registration of the thermal image dataset to a reference visible image, which is difficult or in some cases not possible due to the lack of reference points in the thermal (emissive) signatures, both in the LWIR and MWIR wavelengths.

Recently, it has been shown that the use of the thermal MWIR region allows the design of a dual mode imaging approach^11^, in which a two-step acquisition is performed in the reflective and the emissive domains and where the two datasets can be jointly analyzed in order to obtain information from both the surface and the subsurface of the painting. It is known that, in cultural heritage, reflectance imaging is traditionally performed in the near infrared up to 2.5 ${\upmu }\hbox {m}$ (see the recent review^12^) and that infrared reflectography, initially devoted to unlock hidden features such as drawings in ancient paintings, is being used by museums to support material analysis, especially in large painting^13^. Starting from a simple and fructuous idea, Daffara et al.^11^ have shown that reflectography can be performed in a very good approximation in the thermal mid infrared range, namely, in the MWIR from 3 ${\upmu }\hbox {m}$ to 5 ${\upmu }\hbox {m}$, if a proper non-heating source is matched to the MWIR sensor. The method, named thermal quasi-reflectography (TQR)^14^, is sensitive to the surface features due to the non penetrating character of the MWIR wavelengths when interacting with artworks’ materials^15,16^. The concept was later adopted by the scientific community working on the field of art diagnostics and applied to the study of different kind of artworks, e.g. manuscripts^17^.

In this paper, the proof of concept of frescoes diagnostics by MWIR dual mode imaging method, coupled to a variant of thermal signal reconstruction (TSR)^18^, is shown on an exemplar case study, the Leonardo’s “Monocromo” in the “Sala delle Asse” of the Sforza Castle (Milan, Italy).

**Figure 1:**
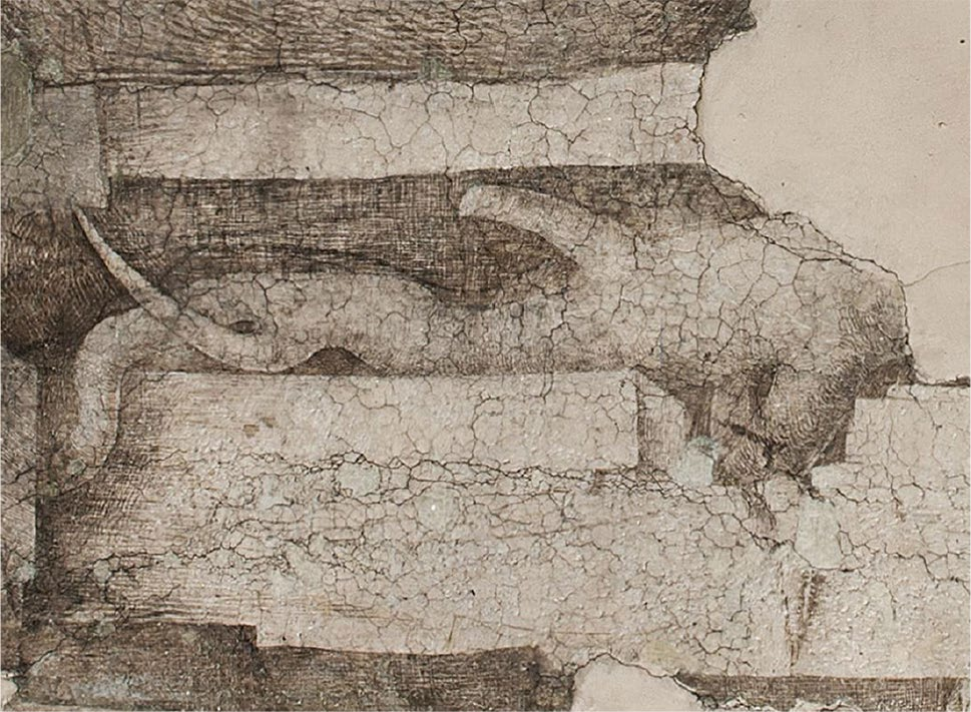
A detail of the “Monocromo” by Leonardo depicting a root.

The “Sala delle Asse” is a large room on the first floor of the north-east great tower of Sforza Castle in Milan (Italy). It got its name from the wooden planks (“Asse”), which once covered the walls. It was an important setting where ambassadors and guest were welcomed by Ludovico Maria Sforza (1452–1508). Leonardo da Vinci realized the pictorial decoration of the room in 1498. However, one year later the city was conquered by the French and a decline period started for the castle: the Sala delle Asse was used as a stable and Leonardo’s painting was covered by lime until the end of the nineteenth century. The monochrome fragments depicting rocks and roots on the walls (Fig. 1) had been attributed to Leonardo during the 1950s restoration. A new great restoration project started in 2013.

Differently from the engineering field, the materials encountered in cultural heritage are characterized by a very complex (and unknown) structure. Each artwork is a unique piece, subjected to material and structural alterations resulting from interactions with the environment as well as from restoration interventions. As a matter of fact, mockups prepared in laboratory, even aged, present several limitations. This is well known by scientists who are applying metrology to art diagnostics, and it is also for this reason that it is a good practice to validate the techniques on-field, working on genuine artworks, with a holistic approach, in collaboration with the restorers. The restoration intervention on the Leonardo’s frescoes, lasting several years, was a great challenge for the research community, which was called to respond with a number of optical techniques^19^. For our research group, it was an unique opportunity to test, develop, and iteratively adapt diagnostic methods to the specific needs of restorers. In a previous paper^11^, we presented the basic idea of dual mode approach with emphasis on optical instrumentation, TQR, and emissivity correction; furthermore, we provided details on image referentiation. Defects mapping was not performed and raw thermograms were enough to present the method. Preliminary attempts to improve defects detection were summarized in a recent conference^20^.

In this Report, we describe as the dual mode imaging in the mid infrared was effectively used, for the first time, to guide the conservators in the delicate phase of consolidation of the detachments during the restoration of the masterpiece^19^. In particular, the underpinning idea was the use of the sharp surface dataset from the thermal reflectance mode for referencing, with unprecedented high spatial accuracy, both the subsurface thermography dataset and the post-processing analysis, thus solving the mosaicking problem as well as localizing the features detected by thermography. Clearly, the method brings great advantages in those cases when the spatial resolution is the key factor for an effective defects mapping, such as the detection of detachments under restoration. The key feature was the coupling of sub-millimeter spatial accuracy on large areas, allowed by the dual mode imaging, with an effective defects detection, allowed by a variant of the TSR method, as described below.

The use of full field infrared techniques for the analysis of detachments in real frescoes, in-situ, is well documented in literature e.g.^6,8,21–23^, from which it is clear that methods for an accurate visible-thermal referentiation are most welcome.

## Results

**Figure 2:**
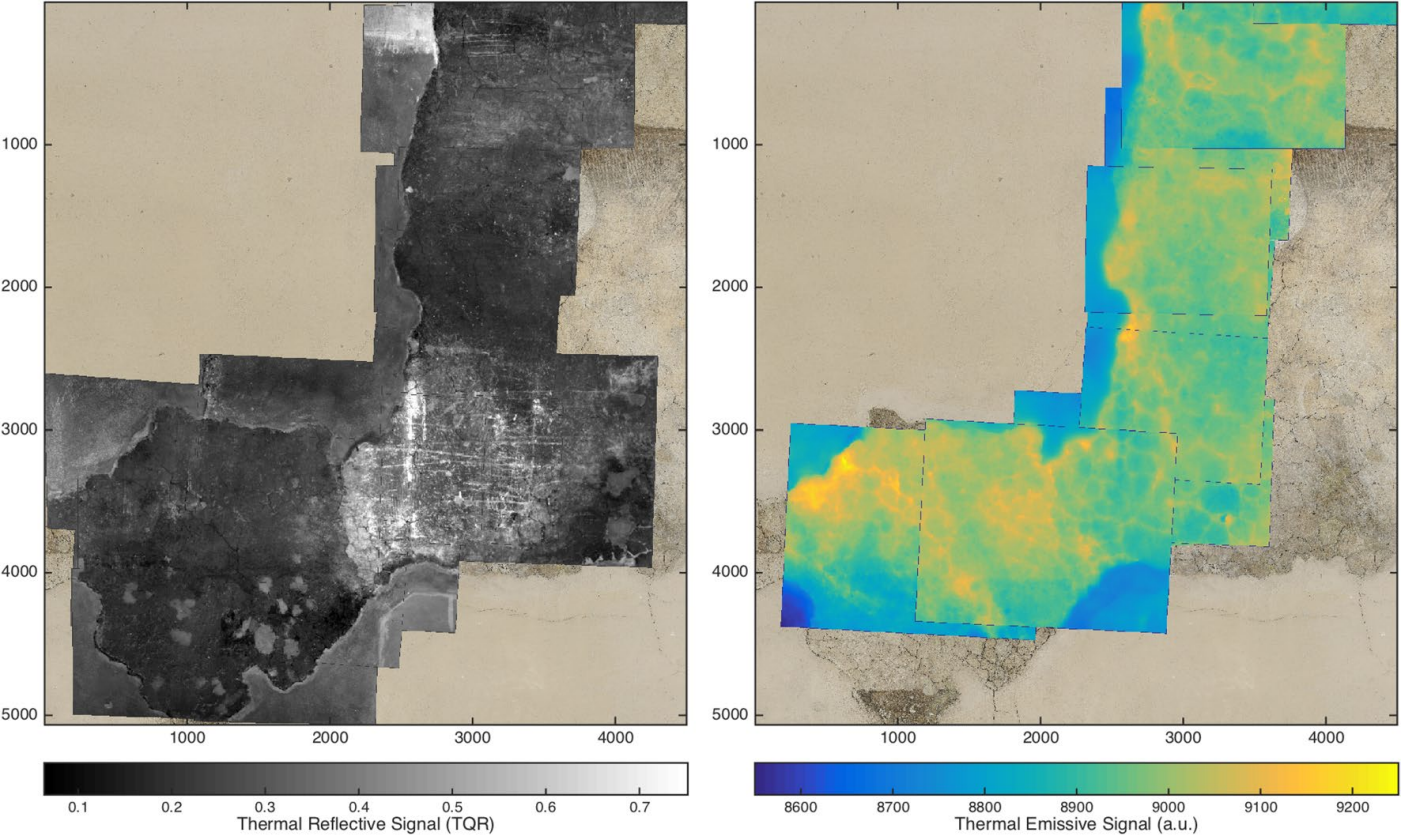
Radiometric mosaics of the TQR frames (left) and of the thermograms (right) registered to the visible, for the central part of the “Monocromo”, approximate dimensions 2.3 m $\times$ 2.5 m, 500 $\upmu \hbox {m}$ pixel size at object plane. High reflectance regions on the lower right zone of the TQR mosaic revealed the presence of crystallised salts.

The main result of this paper shows the full field capability of the dual mode MWIR technique: in Fig. 2 we report the mosaics of the reflectance and the emissive radiometric datasets acquired on the “Monocromo” with sub-millimeter resolution. After spatially registering the TQR frames to a visible orthophoto, a transformation map is obtained and applied to both the dual mode datasets, thus producing two thermal mosaics fully referenced to the visible surface. It is important to underline that, if an ortho-rectified image is not an available option (see the flow chart in Fig. 8 in the “Methods” section), it is the TQR map itself that, being sharp and rich in recognizable surface features (e.g. cracquelure pattern), will play the role of reference “visible” data for the thermography. Moreover, TQR images are easy handled in a mosaic processing, thus applying in a second step the obtained TQR-based transformation map to the thermal sequences.

**Figure 3:**
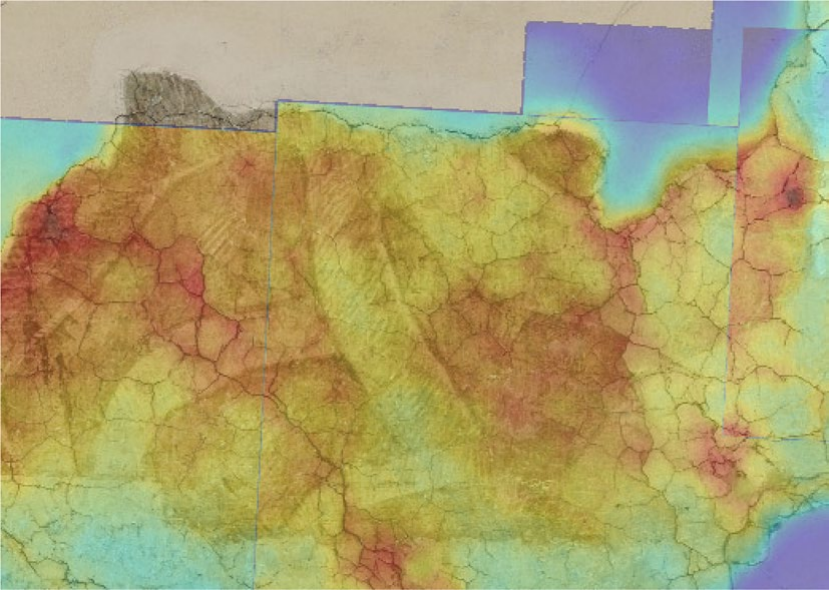
Alpha fusion between registered radiometric thermal image and visible image.

The referentiation of thermography, blurred by thermal diffusion and without visible reference points (markers are not used on frescoes), is done with high accuracy in the reflectance domain; ideally, the process is limited only by the resolution of the MWIR camera at object plane (see Fig. 3). However, it should be said that the simple portable setup with the camera on a photographic tripod is sensitive to external vibrations, and this may induce misalignment in the FOV of the TQR and thermography datasets, especially in such a difficult out-of-laboratory environment as the scaffolding.

**Figure 4:**
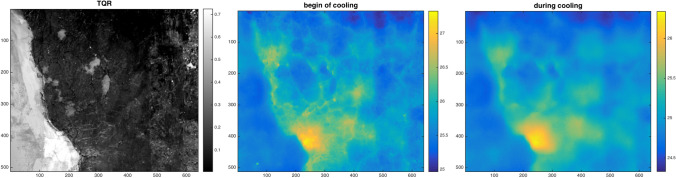
Dual mode MWIR dataset: TQR map and selected thermograms ($^\circ$C) from thermal sequence.

For a frame of interest chosen on the painting mosaic, the Fig. 4 reports the MWIR dual mode results: the TQR map, after MWIR reflectance calibration, and the thermal (emissive) sequence at selected times.

Subsurface defects were localised via the polynomial coefficient maps decomposition by coupling the dual mode paradigma to the TSR processing technique, detailed later. Here, the thermal relaxation part of the thermal signal was decomposed, on a pixel-wise basis, in the log-log domain, using the polynomial approximation of Eq. (5) up to an order of $N=4$, thus generating 5 coefficient maps. Higher orders were also tested but, a low polynomial order was already able to show the desired features as the decomposition was not intended for the reconstruction of the thermal signal in time like in the original Shepard’s technique^18^. Instead, in this work, the $N+1$ maps of coefficients $a_n(x,y)$ are analysed as a set of static intensity images, namely, a set of spatial maps registered to the visible image, for a direct defect localization.

**Figure 5:**
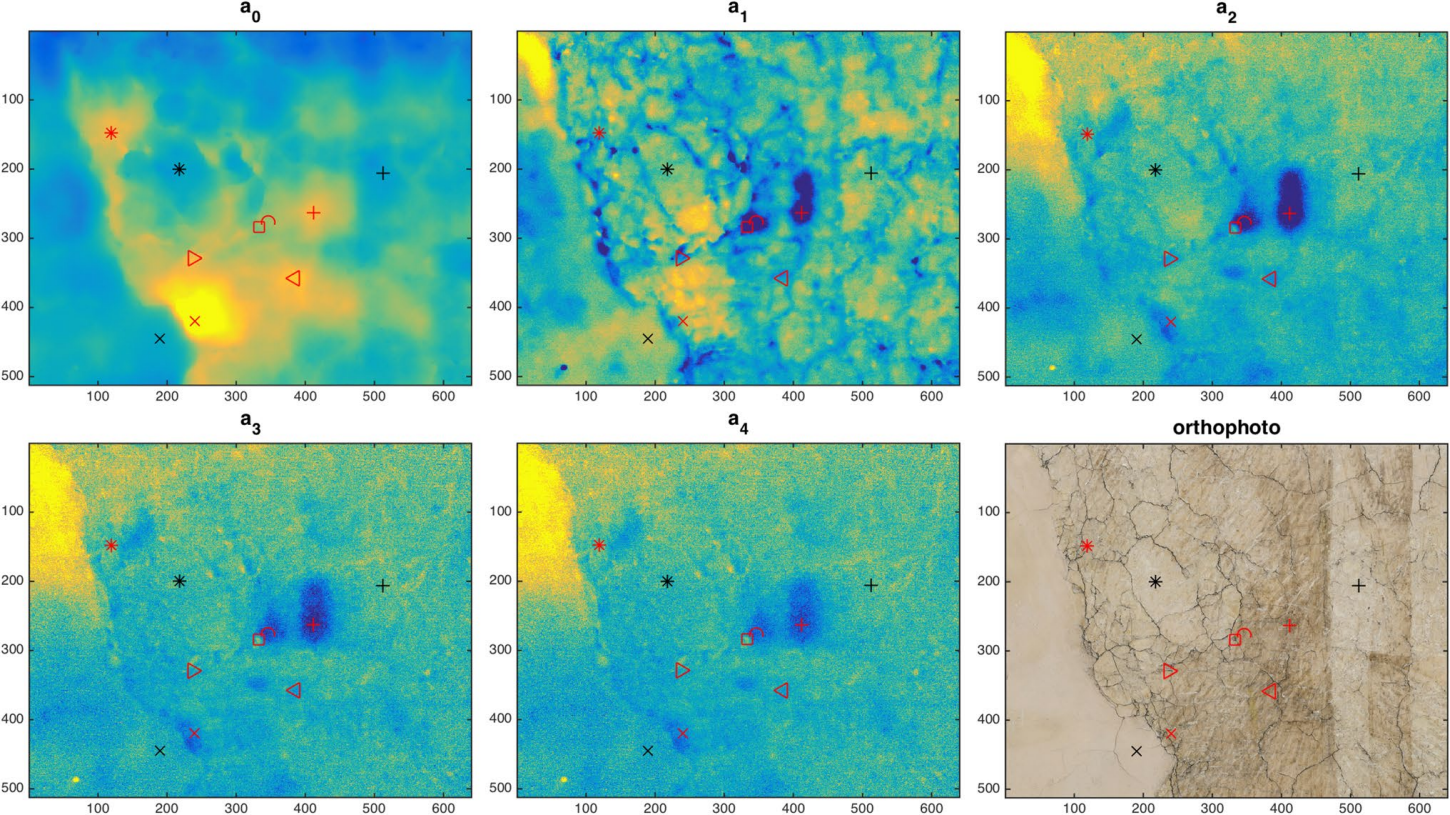
Maps of the polynomial coefficients decomposition with the referencing orthophoto for a selected frame of the mosaic, same 500 $\upmu \hbox {m}$ pixel size at object plane. Red markers indicate the detached regions validated by the restorer, in particular (+) was identified as very serious defect. Among the sound regions marked in black, ($\times$) is located in a part of the wall outside the painting that is well discriminated by TQR (see Fig. 4).

Figure 5 reports, as result, the set of computed polynomial coefficients maps $a_n(x,y)$, for the selected area of interest. A qualitative analysis of the images, together with information available from the restorers, shows the effectiveness of the coefficients maps to reveal the presence of defective features, e.g. cracks pattern, detaches and restoration fillings. The further and important advantage brought by the dual mode method is that the maps are in turn referenced to the visible fresco surface, as well as to the TQR, with same sub-millimeter accuracy.

The heterogeneous nature of the fresco, with the presence of different materials in the surface and in the subsurface stratigraphy, may lead to thermal signatures that make difficult the detection of the defect boundaries. Moreover, the different absorption (in the visible and IR region) of organic and non-organic materials may cause non uniform heating. Thanks to the dual mode approach, an insight on the heterogeneity of surface materials and its reflectivity in the MWIR measuring band (from which, a rough estimation of the variegate emissivity can be obtained^11^) is given by the TQR dataset (see Figs. 2 and 4).

**Figure 6:**
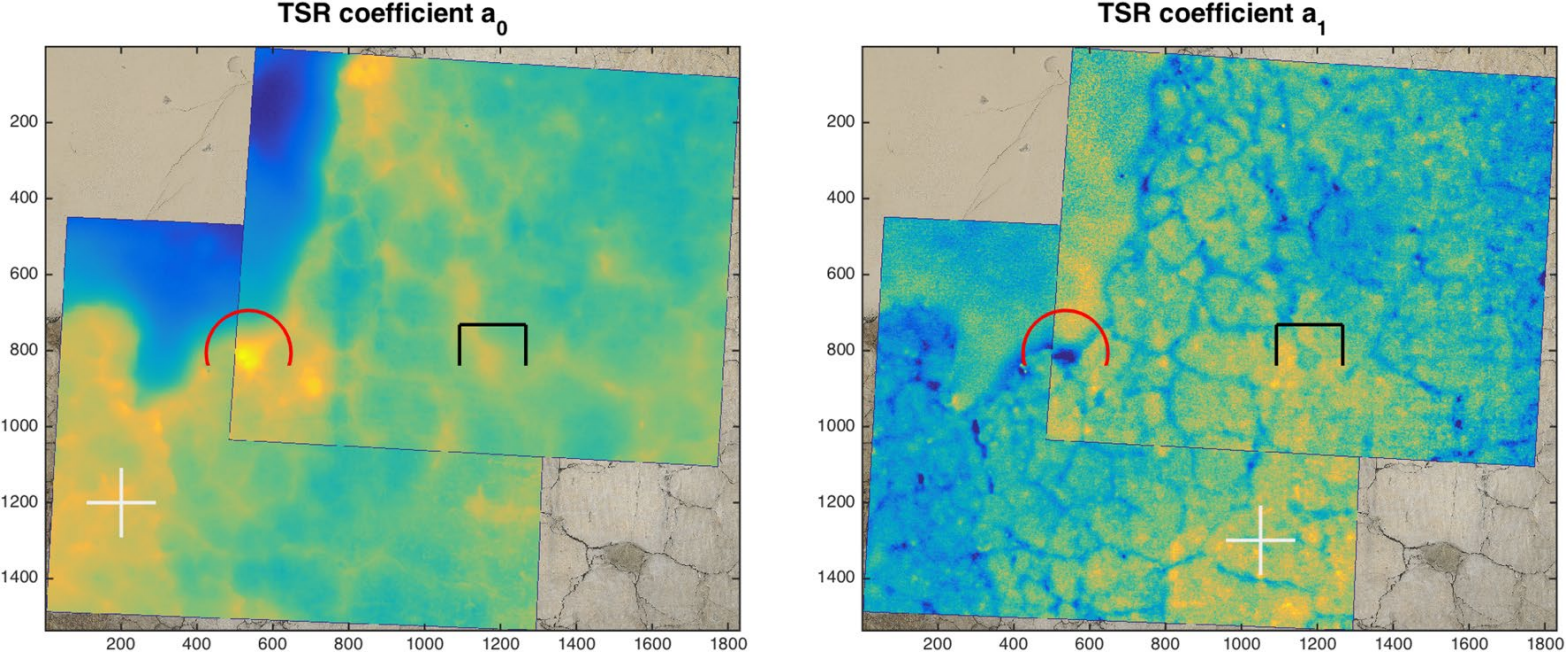
Mosaic of the TSR coefficient maps $a_0$ and $a_1$ registered to the orthophoto for two selected frames. Red marker indicates a significant detachment, black marker an old stucco filling. The sub-millimeter resolution allows the precise mapping of the serious network of cracks through the $a_1$ coefficient. White marker indicates areas of anomalous thermal signature due to the different surface emissivity and heat absorption, which is effectively mapped by the first polynomial coefficient. Emissivity map can be inspected looking at the complementary TQR reflective signal (see Fig. 2).

The possibility of rapidly representing the features of interest by static images, instead of inspecting the sequence of thermograms, is of particular advantage when a large painting is inspected at high resolution, namely, by scanning the wall with several acquisitions, and a considerable number of frames is produced. The dual mode imaging allows the visible referentiation, and then the mosaicking, of the coefficients images thanks to the TQR-based transformation map. Proof of concept of such capability, on selected frames, is given in the results shown in Fig. 6, where the craquelure allows to appreciate the accuracy.

## Discussion

The dual mode MWIR acquisition is performed in two separate, independent steps. The core of the proposed approach is the quasi-reflectography technique that is jointly used with, and then integrated to, the traditional (emissive) thermography. In this work, a portable low cost setup was used to apply a long heat pulse; of course, thermography can be performed with more advanced methods and equipments.

As mentioned, in the dual mode imaging workflow (Fig. 8), the task of processing the thermal data could be faced with a number of advanced techniques available from literature and demonstrated on frescoes, e.g.^4,6,8,22^. Here, the TSR-based method itself is being used only for a qualitative analysis, that is, for mapping the defects without depth characterization. Such specific issues that regard quantitative thermography were out of the scope of this paper, which is focused on the advantages offered by the dual mode acquisition as general workflow.

The long pulse heating allowed to detect the presence of shallow defects within the mural painting, namely the detachments between the finishing surface (fresco layer) and the wall support. The employed excitation energy is in accordance with literature^24^. It should be underlined that high temperature gradients must be always avoided in cultural heritage applications.

Another important issue to discuss is the choice of the MWIR band. Two main reasons have driven the use of a thermal MWIR sensor instead of a LWIR one^14^. The first reason is that the quasi-reflectance approach is not practicable in the LWIR band, because of the contribution of the thermal emission from the object at room temperature on the reflectance signal and the noisier camera. The second reason is that MWIR reflectance has shown to be very informative for frescoes analysis, namely, complementary to the other techniques routinely used. That said, the MWIR dual mode imaging technique is more suitable for inspecting the surface and the subsurface of the fresco layer than the deeper features in the wall support, for which a LWIR camera is most suitable.

## Conclusion

Dual mode imaging allows the scanning of large surfaces thanks to the possibility of creating the mosaic with the images in the reflectance MWIR domain: the resulting transformation map is used for the images acquired in thermal emissive modality as well as for the results of the thermal processing (the defect maps). We integrated the TSR method in the dual mode workflow, showing that the stack of the coefficients maps of the polynomial decomposition of the thermal signal in the log-log domain, using a long pulse stimulus scheme, allowed a reliable defect mapping in a fresco by Leonardo, referenced to the visible surface in a mosaic with high accuracy (500 $\upmu \hbox {m}$ pixel size at object plane). Clearly, this achievement is very important for the applications on mural paintings that require a large number of single acquisition shots. Moreover, it enables the possibility of mapping efficiently the shallow detaches, for which high spatial, sub-millimetric resolution is required. Further important features of the dual mode thermal imaging include the TQR ability to inspect surfaces, e.g. locating restoration materials and saline patinas as well as the possibility to use the quasi-reflectance map to correct emissivity in thermograms analysis.

Finally, for a complete exploitance of the potentialities of the dual mode imaging in the MWIR, it is fundamental using dedicated TQR sources, matched to the sensor and finely tuned in temperature.

## Methods

In the dual mode procedure a single MWIR thermal camera and a set of multiple sources are used to acquire two separate radiometric datasets, one by Thermal Quasi-Reflectography (TQR) and one by active thermography technique. The TQR image (reflectance mode) and the sequence of thermograms (emissive mode), obtained in a fixed geometry and optical setup, are thus spatially registered, apart from minimal errors due to camera vibrations (e.g. due to the presence of scaffolds).

The employed MWIR camera was a FLIR X6540sc with a cooled InSb detector of ${640\times 512}$ sensitive elements, 15 ${\upmu }\hbox {m}$ pixel pitch, and a noise sensitivity of 20 mK. The objective lens was a 25 mm with a FOV of ${22\times 17}^\circ$. The sensor was coupled to a band-pass filter (placed in the internal wheel) to match the nominal camera sensitivity (1.5–5.5 ${\upmu }\hbox {m}$) to the MWIR 3–5 ${\upmu }\hbox {m}$.

The heating sources used for the thermography mode were two 1250 W quartz tungsten halogen lamps. The lamps were positioned symmetrically at a distance of $\sim$ 1 m from the painting, to assure a quasi-constant heat flux density of the excitation over the FOV of the camera. The use of reflector shielding or caisson, was not practical. Moreover, the open space is necessary as large temperature gradient must be absolutely avoid in cultural heritage. Shuttering was needed at the switch off in order to block the reflected signal from the transient lamp cool down, which otherwise was dominating, as the residual emission of an halogen bulb matches the MWIR region. This is also the reason why, in MWIR thermography where the excitation is provided by halogen sources, only the thermal relaxation phase after the heating pulse is analyzed.

The radiation sources for the TQR mode were a custom module, designed from quartz elements (Kanthal alloy filament) and suitable filtering window (sapphire) and described in detail in a previous work^11^; main features were the MWIR spectrum matched to the InSb camera sensor, low heating, and large FOV.

**Figure 7:**
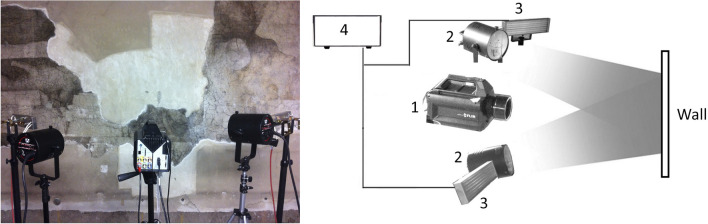
Dual mode measurement setup in situ (left) and sketch of the recording system: (1) IR camera, (2) halogen sources for heating the painting, (3) non-heating TQR sources matched to the sensor, (4) control unit for TQR sources.

The acquisition geometry was setup to obtain a sub-millimetric spatial resolution of ${\sim }\,{0.5}\,{\mathrm{mm}}$, as requested by the restorers. The thermal sequence was acquired after a long pulse heat stimulus of 120 s duration. The acquisition parameters were set on the basis of trials made on a selected area of the painting, with the help of the restorer. A trade-off between high detection capability and low excitation energy was taken into account to preserve the painting from overheating, especially due to the presence of high absorbing organic materials in the surface layer. As mentioned before, in case of layered artworks, which have very heterogeneous thermal properties (diffusivity) and unknown structures, a preventive study on artificial samples in laboratory to enhance the thermal phenomena involved in defects detection may suggest indicative parameters. Figure 7 shows the experimental setup.

### Dual mode MWIR imaging workflow

**Figure 8:**
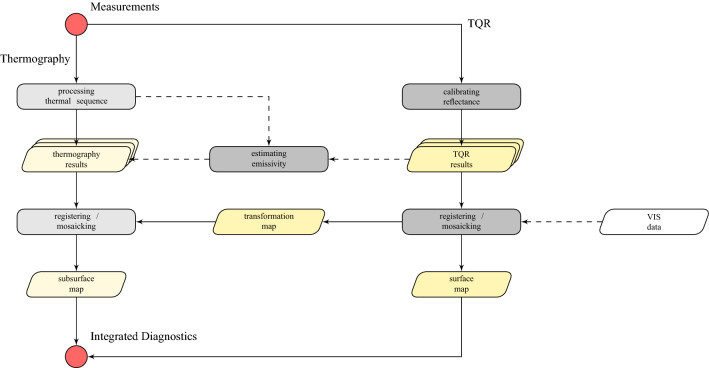
Workflow of MWIR dual mode imaging steps.

The general workflow of the dual mode imaging technique is depicted in the diagram of Fig. 8. Two radiometric image datasets are acquired with the same camera, one in emissive modality (i.e. thermography) and the other in quasi-reflectrography modality (i.e. TQR). The flow chart describes the main concepts of the proposed procedure to obtain an integrated diagnostics. Image processing and visualization is performed in MATLAB^25^ environment.

The set of TQR radiometric images *S*(*x*, *y*) was calibrated using an in-scene standard of certified reflectance value $R_{\mathrm {ref}}$ to obtain the MWIR reflectance map $R^{\mathrm{{TQR}}}(x,y)$, as follows

1 $$\begin{aligned} R^{\mathrm{TQR}} = R_{\mathrm {ref}} \, \frac{S-S^{\mathrm {{BB}}}_{\mathrm {obj}}}{S_{\mathrm {ref}} - S^{\mathrm {{BB}}}_{\mathrm {obj}}}, \end{aligned}$$

where $S_{\mathrm {ref}}$ is the averaged image of the standard and $S^{\mathrm {{BB}}}_{\mathrm {obj}}(x,y)$ is the background signal due to the blackbody emission at object temperature. It has been shown that, for values of reflectance typical for the frescoes’ materials, the accuracy of the quasi-reflectance method is very high, in fact, an estimation of the error induced by the thermal emission in regard to an ideal reflectance measurement *R*(*x*, *y*) is given by^11^

2 $$\begin{aligned} \frac{\Delta S}{S} = \frac{1-R}{R} \, \frac{S^{\mathrm {{BB}}}_{\mathrm {obj}}}{S_{\mathrm {src}}}, \end{aligned}$$

where $S^{\mathrm {{BB}}}_{\mathrm {obj}}/S_{\mathrm {src}}$ is the ratio of the in-band MWIR radiant power emitted from and incident on the object, which is ${\sim }10^{-3}$ for an optimal TQR setup (object at room temperature, MWIR blackbody source picked at ${4}~{\upmu \hbox {m}}$).

Equation (2) clarifies the meaning of the quasi-reflectance approximation. It is evident the importance of using a proper radiation source in mid infrared reflectography, for limiting the heating of the surface and thus performing a reliable measurement of the reflectances.

Regarding the step of thermal sequence processing, a number of techniques are available in literature, qualitative and quantitative, suitable to the specific applications and aims^6,26^. However, the focus in this paper is on the use of dual mode thermal imaging for the localisation of the defects without a quantitative characterization (e.g. depth, thickness). For this reason, we treated the thermal sequence starting from consolidated techniques proposed in the field of cultural heritage, in particular, beside the simple analysis by thermal contrast, the polynomial coefficient map decomposition, detailed later, is used.

### Processing of thermal sequence

The implementation of the module regarding the post-processing analysis of the thermograms in the dual mode workflow represents the core novelty of the work. The method we adopted for analyzing the thermal sequence is based on the TSR technique introduced by Shepard^18^. A comprehensive explanation of the TSR technique and its role in thermographic analysis can be found in literature^27,28^. Initially proposed for pulse thermography, it has shown to be effective also for other excitation schemes, in particular, for the employed long pulse thermography^29^. Hereby, some fundamental concepts are given in relation to the adopted polynomial coefficient map decomposition method.

The behaviour of a homogeneous (defect-free) sample, in which the lateral diffusion components cancel, subjected to a uniform heat pulse ($\sim$ Dirac), is approximated by the one dimensional model, i.e. by the 1-D heat diffusion equation, with the well-known analytical solution for the surface temperature $T(t, z=0)$

3 $$\begin{aligned} \frac{\partial ^2 T}{\partial z^2}-\frac{1}{\alpha }\frac{\partial T}{\partial t} = 0, \end{aligned}$$

4 $$\begin{aligned} \Delta T(t) = \frac{J_0}{e \sqrt{\pi t}}, \end{aligned}$$

with $\alpha$ the thermal diffusivity, *e* the thermal effusivity, and $J_0$ the excitation energy. In the log-log domain, the temperature of a non-defective region decreases linearly with time with a fixed slope of $-1/2$, independent from materials and excitation energy. Bulk discontinuities cause a deviation from the 1D approximation locally, thus allowing the detection of the defect from the anomalous behaviour of the surface temperature field in the relaxing phase. Large and shallow defects are most effectively discriminated due to a minor blurring from diffusion. It is clear that the situation of highly heterogeneous artworks is much more complex. Examples of discontinuities in mural paintings are the void from the detached plaster or the cement from restoration filling.

Starting from the above observations, the thermal signal, from the multilayered mural painting, can be conveniently reconstructed by the coefficients of a polynomial approximation, as follows

5 $$\begin{aligned} \ln (\Delta T(t)) = \sum _{n=0}^{N} a_n[\ln (t)]^n, \end{aligned}$$

6 $$\begin{aligned} \Delta T(t) = \exp \left( \sum _{n=0}^{N} a_n[\ln (t)]^n\right) . \end{aligned}$$

From the polynomial fitting of Eq. (5) the logarithmic derivatives can be analyzed to obtain further information^18^.

For the thermographic analysis, we adopted the long pulse excitation technique because of the advantages of a practical setup for measuring in situ. Long pulse thermography was shown to have optimal performance for the detection of defects in low thermal conductivity materials^30^, as is the case of mural paintings.

In case of long pulse solicitation, i.e. a constant heat pulse *J* of duration $t_p$, the response of a non-defective region in one dimensional approximation can be computed as sequence of impulse responses, i.e. by integration of Eq. (4), finding the following behaviour for the surface temperature in the relaxing phase $t > t_p$

7 $$\begin{aligned} \Delta T(t) = \frac{2J}{e \sqrt{\pi }}\left( \sqrt{t}-\sqrt{t-t_p}\right) , \end{aligned}$$

from which it can be seen that it is still interesting to evaluate the signal decomposition in Eq. (5) originally proposed for the flash pulse. Anyway, in this work, we were not interested in the time reconstruction of the signal. We exploited the log-log decomposition for extracting, pixel-wise, the set of *N* maps of static coefficients $a_n(x,y)$, which turned to be informative of the presence of local anomalies. Moreover, this coefficient image stack was spatially aligned to the TQR and thus referenced to the visible surface thanks to the usual dual mode procedure described below.

The shallow defects are located in the plaster and pictorial layer of the fresco with a variety of sizes and depth from few millimeters to few centimeters. Different kind of inclusions are present, acting as resistive or as conductive discontinuities, for which the thermal conductivity is lower or higher than the bulk material, as the case of the void (air) and the restored (cement) detachments. However, mural paintings have a complex stratigraphy with variety of materials and granular structures, therefore with different (and mostly unknown) thermal and radiative properties. The “defect” has not a rigid classification and quantitative diagnostics is difficult.

### Registering thermograms to the visible and mosaicking

As stated before, we were pushed by the motivation that the referentiation of infrared thermograms to the visible surface or to a rectified-photo is a crucial stage in thermography for frescoes diagnostics and restoration interventions. In fact, the solution offered to the need for high spatial resolution in thermal imaging is the capability of producing a reliable mosaic, i.e. well-referred to the visible, with a large number of small FOVs acquisitions. The steps involved in the dual mode method (Fig. 8) were to perform the visible referentiation and the mosaicking in the reflectance domain (TQR images), and then to apply the same geometric transformation to the emissive domain (thermograms), obtaining the referred thermography mosaic. A further advantage of the procedure, which is presented in this paper, is that the image results of the post-processing analysis, i.e. the defect maps, can be assembled in a referenced mosaic.

**Figure 9:**
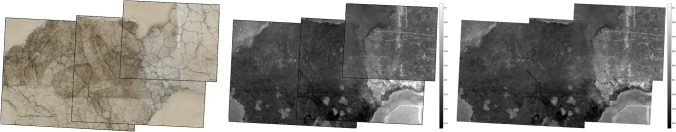
Detail from the “Monocromo”: a root of a tree. From left to right: visible, TQR and the light-balanced TQR obtained with the anisotropic osmosis filter.

The final TQR mosaic was post-processed with an anisotropic version of the *osmosis* filter^31^, a drift-diffusion partial differential equation introduced in^32,33^, effectively able to remove measurements errors in quasi-reflectography data when interpreted as inter-frame change of contrast^34^ (see Fig. 9).
